# pH-dependent secondary structure propensity of the influenza A virus M2 cytoplasmic tail

**DOI:** 10.1007/s12104-020-09937-8

**Published:** 2020-03-10

**Authors:** Jolyon K. Claridge, Faiz Mohd-Kipli, Andrei Florea, Thomas Gate, Jason R. Schnell

**Affiliations:** Department of Biochemistry, University of Oxford, South Parks Road, Oxford, OX1 3QU UK

**Keywords:** Influenza, Matrix protein 2, M2

## Abstract

The cytoplasmic C-terminal tail of the matrix protein 2 (M2) from influenza A virus has a well conserved sequence and is involved in interactions with several host proteins as well as the influenza matrix protein 1 (M1). Whereas the transmembrane domain of M2 has been well characterised structurally and functionally, high resolution information about the distal cytoplasmic tail is lacking. Here we report the chemical shifts of the cytoplasmic tail of M2 and the chemical shift perturbations at low pH and in the presence of membrane mimetics. The cytoplasmic tail residues are mostly disordered but an extended backbone conformation is adopted by the LC3 binding motif and the putative M1 interaction site has partial helical content with a small pH-dependence. The chemical shift assignments provide a basis for further investigations into interactions of the M2 cytoplasmic tail with viral and host cell factors.

## Biological context

Influenza A virus (IAV) is highly infectious and causes a potentially life-threatening illness. IAV is an enveloped virus that buds from host cell membranes and contains three transmembrane proteins: hemagglutinin, neuraminidase, and the matrix protein 2 (M2). M2 is required for genome uncoating upon host cell infection (Helenius 1992), maturation of newly synthesized viral proteins (Hay et al. 1985), and viral budding (Rossman et al. 2010).

**Fig. 1:**
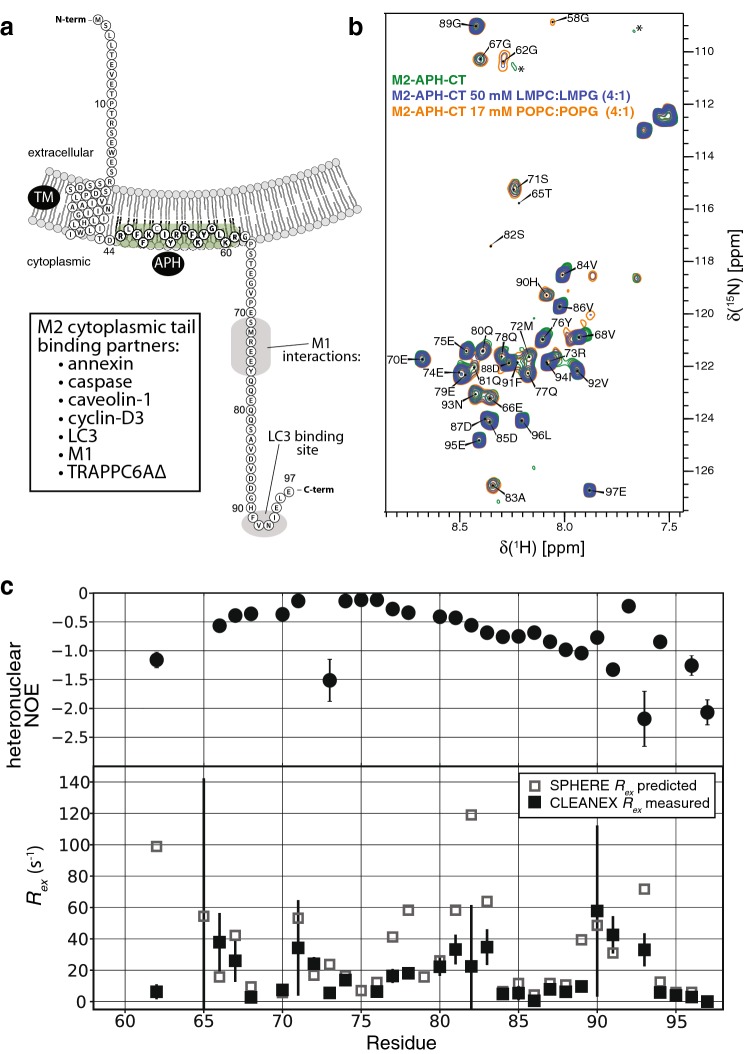
**a** Schematic of full-length influenza M2 with the transmembrane (TM) and membrane-attached amphipathic helix (APH) indicated. Some of the proteins that have been shown to interact with the M2 cytoplasmic tail are listed. The regions responsible for interaction with M1 and LC3 are indicated on the schematic with gray shading. The direction of membrane curvature expected along the axis parallel to the direction of the budding event is shown. **b** Overlay of the amide spectra for M2-APH-CT (residues 44–97) at pH 7.2 by itself (green), in the presence of LMPC:LMPG (4:1) micelles (blue), and in the presence POPC:POPG (4:1) liposomes (orange). The two crosspeaks at ~ 109 ppm and ~ 111 ppm (in ^15^N) and indicated with asterisks are attributed to Gly58 and Gly62 in the absence of membrane mimetics. **c** Top, ^1^H–^15^N heteronuclear NOEs for M2-APH-CT in the presence of POPC:POPG liposomes at pH 7.2. Bottom, amide proton water exchange rates (*R*_ex_) derived from CLEANEX experiments for M2-APH-CT in the presence of POPC:POPG liposomes at pH 7.2. Also shown are the exchange rates predicted from sequence alone using the SPHERE server (Yu-Zhu Zhang, Ph.D. Thesis, University of Pennsylvania, PA, USA.)

M2 is a type I transmembrane protein (Fig. 1a) with a short extracellular N-terminus (~ 25 residues) that adopts a partially extended conformation (Liao et al. 2013; Cho et al. 2015, 2016). The transmembrane domain tetramerizes to form a proton channel targeted by adamantane-based drugs and has been well characterised functionally and structurally (Holsinger and Lamb 1991; Duff and Ashley 1992; Pinto et al. 1992; Schnell and Chou 2008; Stouffer et al. 2008; Cady et al. 2010; Sharma et al. 2010). A membrane proximal cytoplasmic region (residues ~ 46–97) contains a membrane-interacting amphipathic helix (APH; residues ~ 46–62) that is important in lipid raft targeting and virus scission (Schroeder et al. 2005; Rossman et al. 2010; Thaa et al. 2011; Martyna et al. 2017).

In contrast to the rest of the protein, the distal cytoplasmic tail (residues ~ 63–97) has been less well characterised but is a hub for host and viral protein interactions and its removal leads to reduced virus particle production (McCown and Pekosz 2005). Besides interactions with the viral matrix protein 1 (M1) (McCown and Pekosz 2006; Chen et al. 2008), the cytoplasmic tail M2 is also reported to interact with several host proteins including LC3 (Beale et al. 2014), annexin (Ma et al. 2012), cyclin-D3 (Fan et al. 2017), caveolin-1 (Zou et al. 2009), and TRAPPC6A (Zhu et al. 2017), and is cleaved after residue 88 under apoptotic conditions by caspases (Zhirnov and Syrtzev 2009).

Previous solid-state NMR studies are consistent with a flexible M2 cytoplasmic tail, although only limited residue-specific information is available (Liao et al. 2013; Kwon et al. 2015). Here we present essentially complete residue-specific assignments, information on flexibility and solvent accessibility, and the secondary structure propensities for the cytoplasmic tail of M2 under neutral (pH 7.2) and acidic (pH 5.5) conditions.

## Methods and experiments

An M2 gene based on the ‘swine flu’ strain England/H1N1/250 and codon-optimised for *E. coli* expression was purchased from GeneArt and used as the template to generate a M2-APH-CT construct containing the full cytoplasmic tail (residues 44–97) fused to an N-terminal maltose binding protein with a 6-histidine tag. A BL21(DE3) pLysS *E. coli* strain (Invitrogen) transformed with the expression plasmid was grown at 37 °C in LB and induced with 1 mM IPTG at an OD_600_ of 0.6. For labelled growth, the cells were grown to an OD_600_ of 0.6 and then spun down at 2500×*g* for 10 min and resuspended in ¼ volume of M9 minimal media containing 2 g ^15^NH_4_Cl and 4 g ^13^C-glucose per liter. The culture was left shaking at 37 °C for 45 min and then induced with IPTG. The cells were pelleted at 4000×*g* for 15 min at 4 °C then resuspended using a Dounce homogenizer in 60 ml lysis buffer (50 mM Tris-base, 150 mM NaCl, pH 7.8 containing one Complete protease inhibitor tablet (Roche) per 50 ml). 10 mg of lysozyme was added, and the suspension was then passed through a cell disruptor at 30 kpsi at 4 °C. The resulting lysate was spun down at 27,000×*g* for 15 min at 4 °C. The supernatant was passed through a 5 ml HiTrap Ni^2+^-NTA column (GE HealthSciences) at 5 ml per minute and then eluted with an imidazole gradient (from 5 to 300 mM over 50 ml). The fractions containing the protein were pooled, 40 µl of 3C protease (GE) added, and the solution dialysed against 2 l of lysis buffer overnight at room temperature. The resulting cleaved product, containing the non-native N-terminal sequence GPGS in addition to the M2 residues 44–97, was passed back over the Ni–NTA column and the flow-through was concentrated to 500 µl and passed over a Superdex S75 10/300 column (GE HealthSciences) at 0.75 ml per minute in NMR buffer (20 mM NaPO_4_, 20 mM Na acetate, 137 mM NaCl, 2.7 mM KCl, and pH 7.2 or pH 5.5). The fractions containing M2-APH-CT eluted between 13 and 13.5 ml. The samples were concentrated to 150–300 µM for NMR experiments.

Liposomes containing POPC:POPG (4:1) were prepared by dissolving lipids into chloroform:methanol at a ratio of 5:1 and then thin-filming the lipid mixture under a nitrogen gas stream. Films were then re-dissolved in chloroform and a new thin-film made under nitrogen. Any remaining chloroform was removed by vacuum for 2 h. The film was resuspended into NMR buffer at pH 5.5 or pH 7.2 and several freeze–thaw cycles used to form liposomes. In order to homogenise liposome sizes the sample was extruded through a 0.2 µm membrane.

NMR experiments were carried out at 37 °C. Resonances were assigned using BEST versions of 3D HNCA, HNCOCACB, and HNCO spectra (Schanda et al. 2006) at 600 MHz (^1^H) collected on ^13^C-^15^ N labelled protein in the presence of 50 mM LMPC:LMPG (4:1) micelles. T_1_, T_2_, heteronuclear NOE, CLEANEX (Hwang et al. 1998) experiments were collected at 600 MHz (^1^H) using ^15^N-labelled protein at pH 7.2 in the presence of POPC:POPG (4:1) liposomes. NMR data were processed using NMRPipe (Delaglio et al. 1995) and analysed using CcpNmr Analysis (Vranken et al. 2005). Proton chemical shifts were referenced to DSS and ^15^N and ^13^C chemical shifts were referenced indirectly to proton. Secondary chemical shifts were analysed with TALOS-N (Shen and Bax 2013). The backbone chemical shift assignments of M2-APH-CT at pH 7.2 and pH 5.5 have been deposited in the BioMagResBank (https://www.bmrb.wisc.edu/) with accession numbers 50113 and 50127.

## Assignments and data analyses

An HMQC spectrum of M2-APH-CT is shown in Fig. 1b. Complete backbone and sidechain ^13^Cβ assignments were made for all nonprolines in residues 65–97. Backbone amide resonances for residues 44–64 were largely missing due to chemical exchange broadening. Partial assignments could be made for residues Gly58 and Gly62, which are within the APH. HMQC spectra of the M2-APH-CT construct were acquired in the presence of 50 mM LMPC:LMPG micelles (4:1) or POPC:POPG liposomes (4:1; 17 mM total lipid) at pH 7.2 (Fig. 1b). The detectable amides of the APH residues Gly58 and Gly62 were perturbed upon addition of the membrane mimetics. By contrast, none of the detectable resonances for residues outside of the APH were perturbed upon addition of either micelles or liposomes at pH 7.2 or pH 5.5, consistent with there being no interactions of the distal cytoplasmic tail with membranes.

^1^H–^15^N heteronuclear NOEs and CLEANEX measurements of amide-water proton exchange rates were recorded in the presence of POPC:POPG (4:1) liposomes at pH 7.2. All measured heteronuclear NOEs were negative (Fig. 1c, top), indicating that the distal cytoplasmic tail was highly dynamic throughout. Most exchange rates were comparable to the theoretical values but some protection was observed in residues 62, 77–78, and 81–83 (Fig. 1c, bottom).

**Fig. 2:**
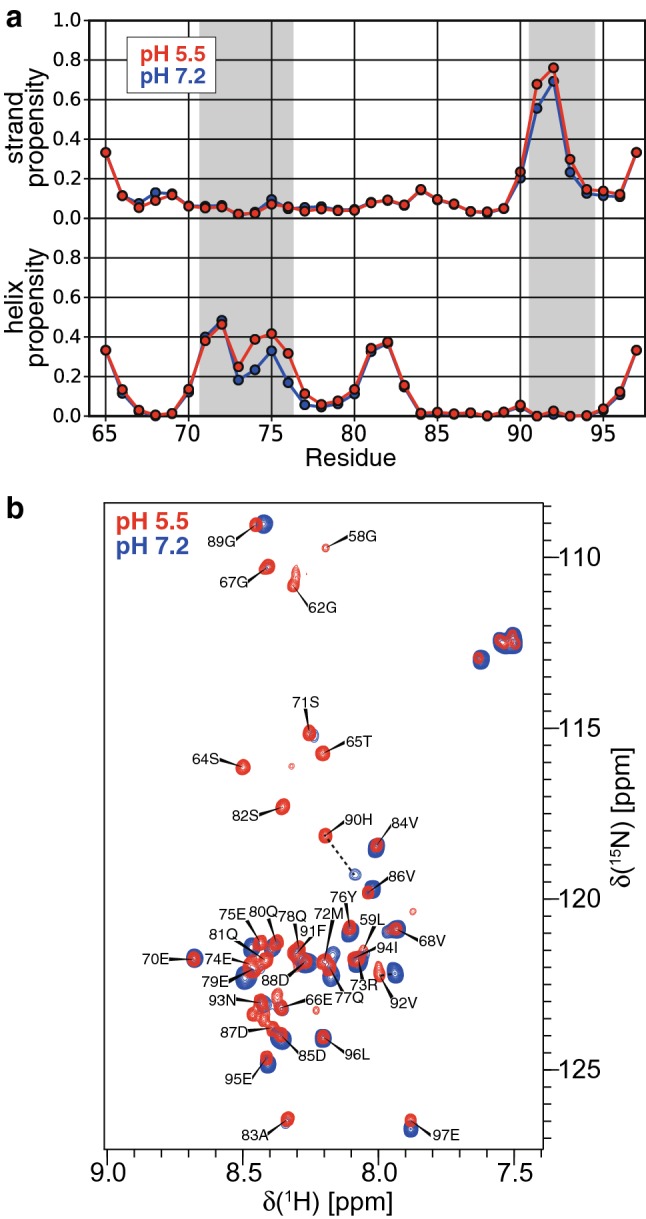
**a** Helix and strand propensities of M2-APH-CT at pH 7.2 and pH 5.5 as determined from chemical shifts using TALOS-N. The regions 70–72 and 91–94 are shaded to indicate the presence of secondary structure in the binding sites for M1 and LC3, respectively. **b** Overlay of the amide spectra of M2-APH-CT at pH 5.5 and pH 7.2 in the presence of 50 mM LMPC:LMPG (4:1). The chemical shift perturbations of H90 and V92 are emphasised with dotted lines. Both spectra were recorded at 310 K

The strand and helix propensities were calculated from secondary chemical shifts (Fig. 2a). An extended, strand conformation was observed in residues 91–94, consistent with sequence-based predictions and its ability to bind LC3 in an extended conformation (Beale et al. 2014). Whereas sequence-based algorithms strongly predicted a helix in residues 71–76, only partial helical content was observed in these residues based on secondary chemical shifts at pH 7.2. Residues 81–82 were also found to be weakly helical.

The effect of pH on the backbone amide M2-APH-CT spectrum in the presence of LMPC:LMPG micelles and the secondary structure are shown in Fig. 2. The largest backbone amide chemical shift perturbations were observed near His90, which is the only histidine in the construct. Additional peaks appeared at low pH which we attribute to APH residues that were not observable at higher pH due to chemical exchange; however these amides could not be assigned even at low pH due to weak or absent correlations. The strand content of 91–94 and the helical content of residues 73–77 slightly increased at pH 5.5. Although the pH-dependent changes observed are small, residues 71–73 and 74–76 are known to have large effects on virus budding and M1 incorporation into virions (Chen et al. 2008) and structural changes here may fine-tune the pH-regulation of the M1-M2 interaction during the viral life cycle.

The very high degree of flexibility in the M2 cytoplasmic tail is consistent with its role as a hub for protein–protein interactions. The M2 cytoplasmic tail is also involved in membrane curvature and fission, and steric pressure from the large hydrodynamic radii of disordered cytoplasmic tails has been shown to facilitate membrane curvature (Busch et al. 2015) and fission (Snead et al. 2017). The strongly negative charge of the M2 cytoplasmic tail would be expected to further increase this steric effect through electrostatic repulsion.
